# Genetic Parameter Estimates for Metabolizing Two Common Pharmaceuticals in Swine

**DOI:** 10.3389/fgene.2018.00040

**Published:** 2018-02-13

**Authors:** Jeremy T. Howard, Melissa S. Ashwell, Ronald E. Baynes, James D. Brooks, James L. Yeatts, Christian Maltecca

**Affiliations:** ^1^Department of Animal Science, North Carolina State University, Raleigh, NC, United States; ^2^Department of Population Health and Pathobiology, Center for Chemical Toxicology and Research Pharmacokinetics, North Carolina State University, College of Veterinary Medicine, Raleigh, NC, United States

**Keywords:** swine, pharmacogenomics, heritability, fenbendazole, flunixin meglumine

## Abstract

In livestock, the regulation of drugs used to treat livestock has received increased attention and it is currently unknown how much of the phenotypic variation in drug metabolism is due to the genetics of an animal. Therefore, the objective of the study was to determine the amount of phenotypic variation in fenbendazole and flunixin meglumine drug metabolism due to genetics. The population consisted of crossbred female and castrated male nursery pigs (*n* = 198) that were sired by boars represented by four breeds. The animals were spread across nine batches. Drugs were administered intravenously and blood collected a minimum of 10 times over a 48 h period. Genetic parameters for the parent drug and metabolite concentration within each drug were estimated based on pharmacokinetics (PK) parameters or concentrations across time utilizing a random regression model. The PK parameters were estimated using a non-compartmental analysis. The PK model included fixed effects of sex and breed of sire along with random sire and batch effects. The random regression model utilized Legendre polynomials and included a fixed population concentration curve, sex, and breed of sire effects along with a random sire deviation from the population curve and batch effect. The sire effect included the intercept for all models except for the fenbendazole metabolite (i.e., intercept and slope). The mean heritability across PK parameters for the fenbendazole and flunixin meglumine parent drug (metabolite) was 0.15 (0.18) and 0.31 (0.40), respectively. For the parent drug (metabolite), the mean heritability across time was 0.27 (0.60) and 0.14 (0.44) for fenbendazole and flunixin meglumine, respectively. The errors surrounding the heritability estimates for the random regression model were smaller compared to estimates obtained from PK parameters. Across both the PK and plasma drug concentration across model, a moderate heritability was estimated. The model that utilized the plasma drug concentration across time resulted in estimates with a smaller standard error compared to models that utilized PK parameters. The current study found a low to moderate proportion of the phenotypic variation in metabolizing fenbendazole and flunixin meglumine that was explained by genetics in the current study.

## Introduction

The regulation of drugs used to treat livestock has received increased attention due to animal welfare concerns, food safety, and implications of antibiotic resistance on human health (Landers et al., 2012). Furthermore, in the context of animal derived foods, Lin et al. (2016) recently addressed issues relating to the current practice of establishing drug tolerance and withdrawal times for unhealthy animals and its inability to address the variation across animals and how disease status impacts drug clearance. To gain a better understanding of the variation and impact of genetic variability on swine drug metabolism, a resource population was created and initially described in Howard et al. (2014). Within this resource population, it was shown by Howard et al. (2014) that differences in pharmacokinetic (PK) parameters exist across four major swine breeds (i.e., Duroc, Hamphsire, Yorkshire, and Landrace) and sex. This was further verified at the genomic level based on gene expression differences for genes previously shown to play a role in drug metabolism, which include *SULT1A1* and *CYP2E1* for flunixin meglumine and *SULT1A1* for fenbendazole (Howard et al., 2015). Furthermore, across the liver transcriptome, multiple cytochrome P450 (CYP1A1, CYP2A19, and CYP2C36) genes displayed different transcript levels across animals administered a drug vs. untreated animals (Howard et al., 2017). Gene expression differences highlighted that genetic variation exists in how pigs metabolize drugs. Nonetheless, the amount of variation in the parameters that describe the rate at which a drug is metabolized that is attributable to genetics is currently not well-understood.

The resource population initially investigated four different drugs that involve both phase 1 and 2 drug reactions and are commonly used to treat livestock. A preliminary investigation that is described in Howard et al. (2014) narrowed down the drugs to two, fenbendazole and flunixin meglumine, that encompass different drug metabolism reactions and a portion of the PK parameters displayed significant breed and/or sex differences. Fenbendazole is a antihelmintic drug that is widely used in swine due to its broad-spectrum activity. It is primarily administered as a feed additive. Fenbendazole is predominantly oxidized to its fenbendazole sulfoxide metabolite, oxfendazole (active metabolite) which is then further metabolized to its sulfone metabolite, fenbendazole sulphone (inactive metabolite) via the mixed function oxidase system (Petersen and Friis, 2000; Howard et al., 2014). Lastly, fenbendazole has a zero-day labeled withdrawal time. Flunixin meglumine is used for the control of pyrexia associated with swine respiratory disease and has a labeled withdrawal time of 12 days (Howard et al., 2014). Flunixin meglumine inhibits cyclo-oxygenase, which decreases prostaglandin synthesis. Flunixin is predominantly oxidized by the mixed function oxidase system to 5-hydroxy flunixin (Buur et al., 2006). For both drugs, our recent work suggest that *CYP1A1* and *CYP2E1* are primarily involved in phase one metabolism and phase two often involves sulfate (*SULT1A1*) and possibly glucuronide conjugation. (Howard et al., 2015, 2017). The objective of the current study is to determine the amount of phenotypic variation in fenbendazole and flunixin meglumine drug metabolism due to genetics.

## Materials and methods

This study was approved by the NCSU Institutional Animal Care and Use Committee (IACUC) and all experiments were performed in accordance with relevant guidelines and regulations.

### Animals and experimental design

The resource population that was utilized to estimate genetic parameters was developed to investigate the phenotypic and genetic variability related to drug metabolism in swine (Howard et al., 2014, 2015, 2017). The animals consisted of crossbred female and castrated male nursery pigs (*n* = 198) housed in individual pens (0.9 × 1.8 m) at a room temperature of 17–24°C. Only 32 pens were available at a given time, therefore the animals were spread across nine batches. All animals utilized in the study were fed an *ad libitum* standard commercial corn and soybean meal based diet throughout the study.

The sires of the pigs were registered National Swine Registry sires mated to sows (Yorkshire X Landrace) at the North Carolina State University Swine Education Unit. A subset of the female and castrated male offspring produced by each mating pair was selected for the study. The sires utilized were either purebred Duroc (*n* = 5), Hampshire (*n* = 4), Landrace (*n* = 6), or Yorkshire (*n* = 5). Not all sires were utilized within each batch, although 11 out of the 20 sires were utilized across more than one batch. As a result, some degree of confounding between sire and batch does exist. The average (min-max) number of progeny across sires was 9.9 (4–24). Across all batches except batch 6, each breed and sex class was represented. For batch 6, the Yorkshire breed was not represented due to fertility issues.

The experimental design for batches 1–4 was a random crossover design and is described in detail by Howard et al. (2014). Briefly, two consecutive drug treatments were assigned to an individual. The drugs utilized were paired so that fenbendazole was administered followed by flunixin meglumine or vice versa. Drug treatments were administered 1–2 weeks apart in order to minimize any drug carryover effect and this effect was found to be insignificant in Howard et al. (2014). In batches 5–9 only a single drug treatment of either fenbendazole or flunixin meglumine was administered to an individual. Across all of the batches the number of animals administered flunixin meglumine or fenbendazole was balanced across breed and sex, with the exception of a greater number of Yorkshire sired animals receiving flunixin meglumine in batch 9 to equalize Yorkshire flunixin meglumine treatments across all batches. Summary statistics on the number of animals and average weight prior to drug administration within each batch is outlined in Table 1. For animals in the crossover design (i.e., batches 1–4), an animal was weighed before each drug administration to ensure all animals received the same dose.

**Table 1:** Descriptive statistics across batches.

**Batch**	**Animals**	**Mean ± *SD* weight**	**Drug administered**^a^	
			**FBZ**	**FLU**
1	7	34.15 ± 4.67	6	2
2	12	54.85 ± 11.13	9	10
3	15	31.66 ± 5.18	13	12
4	20	40.66 ± 5.88	17	18
5	29	26.03 ± 4.14	16	13
6	29	26.82 ± 3.78	13	16
7	27	33.67 ± 4.82	13	14
8	28	33.13 ± 5.29	15	13
9	31	34.06 ± 5.11	12	19

a *Refers to drug and FBZ, fenbendazole; FLU, flunixin meglumine*.

### Dosing and blood collection

Fenbendazole and flunixin meglumine were administered intravenously (IV). The IV administration was utilized in order to remove inter- and intra-individual variability observed with extravascular administration routes (Petersen and Friis, 2000; Pairis-Garcia et al., 2013). The description of the dosing and blood collection protocol is described by Howard et al. (2014). Fenbendazole (0.4 g; Sigma-Aldrich, Co.) was administered at an average single intravenous dose of 1 mg/kg. Banamine Injectable Solution (50 mg/mL; each mL contains 50 mg flunixin as the meglumine salt; Merck Animal Health, Summit, NJ, USA) was used to administer an average single intravenous dose of 3 mg/kg of flunixin meglumine. Blood samples (~5 mL) were collected at 0, 0.5, 1, 2, 3, 4, 6, 12, 24, and 48 h postdrug administration for fenbendazole and at 0, 0.5, 1, 2, 3, 4, 8, 12, 24, and 48 h postdrug administration for flunixin meglumine. Following blood collection, the parent drug and metabolite concentrations in the plasma across individuals and time points were measured. The metabolites for fenbendazole and flunixin meglumine were oxfendazole and 5-hydroxy flunixin, respectively. The protocol to determine the parent drug and metabolite concentrations is outlined in Howard et al. (2014).

### Statistical models

Heritability estimates for drug metabolism parameters were obtained based on traditional PK parameters or actual parent drug and metabolite concentrations across time. The latter heritability represents the heritability of the concentration of drug in the plasma across time, while the former represents the heritability of parameters that describe the concentration of drug in the plasma across time. The PK parameters are the traditional metric utilized in veterinary medicine to describe the rate at which the parent drug and metabolite are circulating in the plasma and are the ones that have been traditionally employed in pharmacogenetics analyses. Given the relatively large resource population in the current study, more complex random regression models were utilized to better describe the concentration of the parent drug or metabolite across time.

### Pharmacokinetic parameter model

The PK parameters were calculated utilizing a traditional non-compartmental analysis of drug plasma concentration vs. time profiles. The analysis was conducted using the pharmacokinetic modeling Phoenix software (version 1.1; Pharsight, Cary, NC, USA). The PK parameters calculated for the parent drug included: area under the plasma concentration-time curve from time zero to infinity (AUC_0 → ∞_; h^*^μg/mL), clearance (Cl; L/h/kg), half-life (T_1/2_; h), mean residence time (MRT; h), and volume of distribution at steady state (Vd_ss_; L/kg). The PK parameters calculated for the metabolite included: AUC_0 → ∞_, peak concentration (C_max_; μg/mL), and time at which maximum concentration occurs (T_max_; h). Summary statistics on the PK parameters across both drugs for the parent drug and metabolite are outlined in Table 2. For each PK parameter, the following mixed linear sire model was utilized to estimate genetic parameters within each drug for the parent drug and metabolite:

$$\begin{array}{l} Y_{ijklm}=\;\mu +\;sex_{i}+breed_{j}+batch_{k}+sire_{l}+{\;}^{e_{ijklm}}{/}_{w_{ijklm}}, \end{array}$$

where *Y*_*ijklm*_ represented the PK parameter, μ was the average PK parameter, *sex*_*i*_ was the fixed effect of *i*^*th*^ sex, *breed*_*j*_ was the fixed effect of the *j*^*th*^ breed of the sire, *batch*_*k*_ was the random effect of the *k*^*th*^ batch, sire_l_ was the random effect of the *l*^*th*^ sire and *e*_*ijklm*_ was the random residual with a homogenous variance structure. The residuals were weighted according to the R-squared value of the model for each individual animal. The batch and sire effects were assumed to follow a normal distribution that was identically and independently distributed. The genetic relationships among sires was investigated by tracing back a pedigree for all sires and was found to be near zero in all cases. Therefore, the sires were assumed to be unrelated. Lastly, the impact of body weight, which was used to determine the dose, was investigated and found to be insignificant (*P* > 0.05) across PK parameters and therefore was not included in the final model. The analysis was conducted using the ASReml program (Gilmour et al., 2009). The sire variance estimate was transformed algebraically into direct additive genetic effects $\left(\sigma_{a}^{2}\right)$ by multiplying the sire variance by four. The heritability (*h*^2^) was estimated according to:

$$\begin{array}{l} h^{2}=\;\frac{\sigma_{a}^{2}\;}{\sigma_{sire}^{2}+\sigma_{batch}^{2}+\sigma_{e}^{2}\;}\;. \end{array}$$

The precision of the heritability estimate, indicated by its standard error, was evaluated in order to determine if the estimation error surrounding the heritability (i.e., h^2^ ± *SE*) contained the value of zero, meaning no genetic variation for the trait.

**Table 2:** Observed average (± *SD*) pharmacokinetic parameters^a^ by Drug.

**Drug**	**Compound^b^**	**T_1/2_ (h)**	AUC_0 → ∞_ (h^*^μg/mL)	**MRT (h)**	**Cl (L/h/kg)**	**Vd_ss_ (L/kg)**	**T_max_ (h)**	**C_max_ (μg/mL)**
FBZ	Parent	15.14 ± 12.43	5.62 ± 4.67	15.33 ± 14.7	0.27 ± 0.22	2.93 ± 1.85	–	–
	Metabolite	–	7.02 ± 1.97	–	–	–	3.28 ± 0.69	0.47 ± 0.09
FLU	Parent	6.62 ± 2.32	28.04 ± 8.27	3.85 ± 1.46	0.12 ± 0.04	0.43 ± 0.18	–	–
	Metabolite	–	0.83 ± 0.48	–	–	–	0.51 ± 0.08	0.21 ± 0.10

a The PK parameters were half-life (T_1/2_; h), clearance (Cl; L/h/kg), area under the plasma concentration-time curve from time zero to to infinity (AUC_0 → ∞_; h^*^μg/mL), mean residence time (MRT; h), volume of distribution at steady state (Vd_ss_; L/kg), peak concentration (C_max_; μg/mL), and time at which maximum concentration occurs (T_max_; h).

b *Refers to drug and FBZ, fenbendazole; FLU, flunixin meglumine*.

### Random regression model based on concentration across time

**Figure 1:**
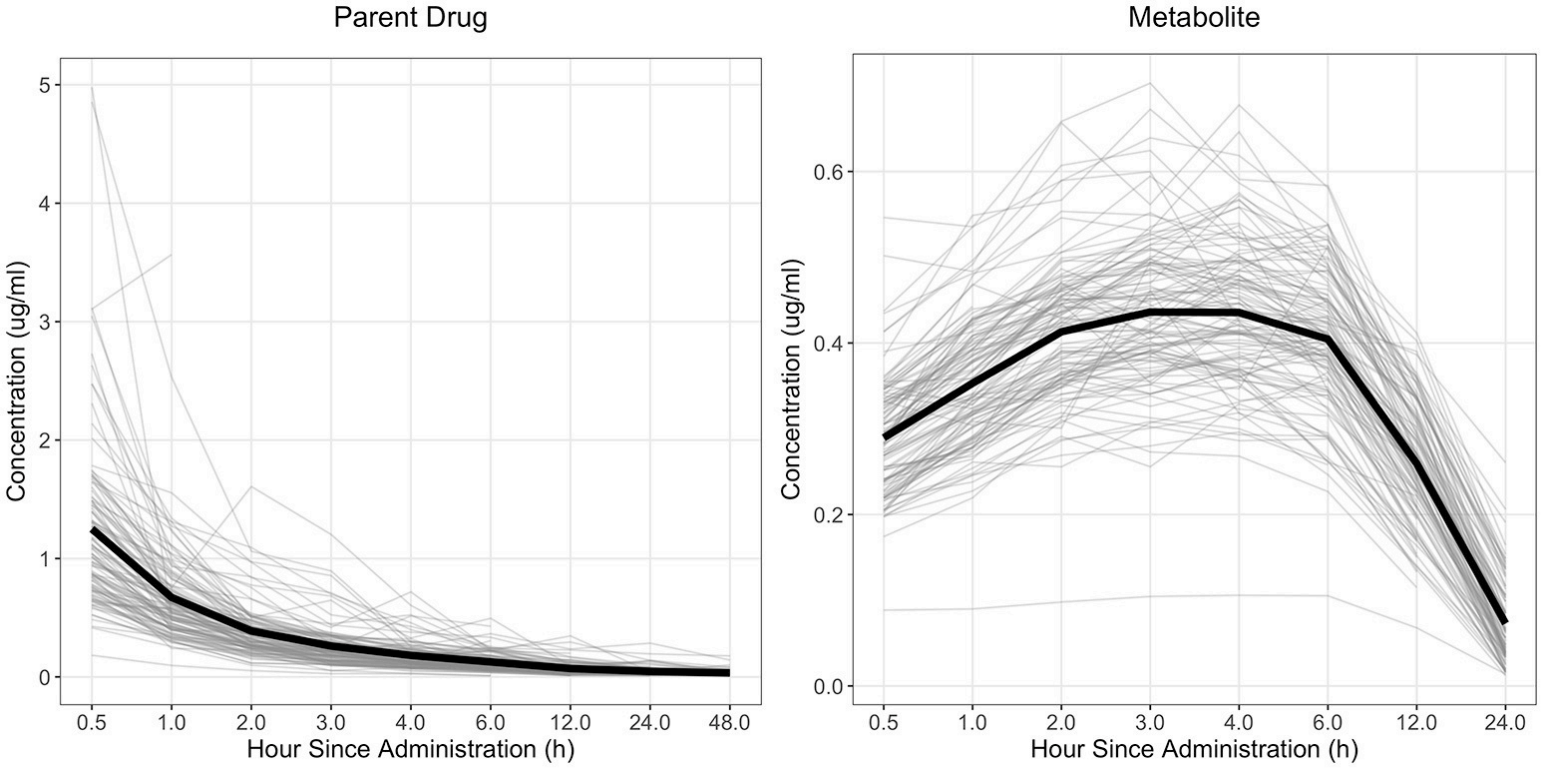
Observed concentration of the parent drug and metabolite across time for fenbendazole. Light gray lines represent individual animals and the solid black line represents the mean across all animals.

**Figure 2:**
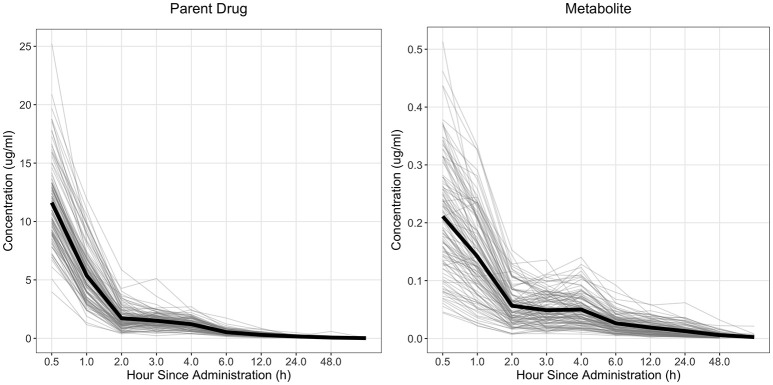
Observed concentration of the parent drug and metabolite across time for flunixin meglumine. Light gray lines represent individual animals and the solid black line represents the mean across all animals.

The drug concentration curve heritability for the parent drug and metabolite within each drug was estimated utilizing plasma concentrations across time for each individual. The raw drug concentration across time for the parent drug and metabolite is summarized in Figures 1, 2 for fenbendazole and flunixin meglumine, respectively. A random regression model was utilized to describe the plasma concentration population mean across time. Sire-specific plasma concentrations across time were represented as deviations from the population mean. The function used to model the concentration profile was an orthogonal Legendre polynomial. As illustrated in Figures 1, 2 the concentration vs. time profiles were different across drugs and within a drug across parent drug and metabolite. As a result, models describing the parent drug and metabolite across both drugs were parameterized slightly different. Within the parent drug and metabolite analysis for each drug, the final model was chosen based on a likelihood ratio test between a reduced and more complex model. The regression of body weight on the parent drug or metabolite concentration was insignificant (*P* > 0.05) for the parent drug and metabolite across both drugs and therefore was not included in the final model. The general mixed linear sire model within each drug for the parent drug and metabolite was parameterized as follows:

$$\begin{array}{l} Y_{ijklmnt}=\;\overset{nf}{\underset{n=1}{\sum }}\phi_{mtn}\beta_{n}+sex_{i}+breed_{j}+batch_{k}\\ +\overset{nr}{\underset{n=1}{\sum }}\phi_{mtn}sire_{\ln}+e_{ijklmnt}, \end{array}$$

where *Y*_*ijklmnt*_ represents the concentration of the parent drug or metabolite, ϕ_mtn_ is Legendre polynomial_n_ for the *m*^*th*^ animal at the *t*^*th*^ hour since the drug was administered, β_n_ are the fixed Legendre polynomial regression coefficients, *sex*_*i*_ was the fixed effect of *i*^*th*^ sex, *breed*_*j*_ was the fixed effect of the *j*^*th*^ breed of the sire, *batch*_*k*_ was the random effect of the *k*^*th*^ batch, *sire*_*ln*_ are the random Legendre polynomial regressions for the *l*^*th*^ sire and *e*_*ijklmnt*_ was the random residual with a heterogeneous variance structure by *t*^*th*^ hour since the drug was administered. The order of the Legendre polynomial for the fixed effect (nf) was 6 for fenbendazole across both compounds and 7 for flunixin meglumine across both compounds. The Legendre polynomial for the random sire regression effect (nr) included only the intercept (i.e., order = 0) for all models except for the fenbendazole metabolite, which included an intercept and slope (i.e., order = 1). Lastly, across both compounds, the residual covariance structure was independent across time points within an individual and across animals for fenbendazole and had an autoregressive 1 (AR1) structure within an animal and independent across animals for flunixin meglumine.

Similar to the PK heritability analysis, the sire variance estimate was transformed algebraically into direct additive genetic effects $\left(\sigma_{a}^{2}\right)$ by multiplying the sire variance by four. For the models that only included an intercept (i.e., flunixin meglumine parent drug and metabolite and fenbendazole parent drug) the sire variance was constant across time and therefore the previous heritability formula was utilized; although it should be noted that the residual variance was heterogeneous across time, resulting in the heritability changing across time points. For the fenbendazole metabolite model, a sire (co)variance matrix (**S**) of dimension 2 by 2 (i.e., intercept and slope) was generated for the sire effect. The sire variance for time point_t_ $(\sigma_{sire\_t}^{2})\;$ was obtained as:

$$\sigma_{sire\_t}^{2}=t_{t}S{t^{\prime }}_{t},$$

where **t**_t_ is equal to the t^th^ row vector of ϕ and **S** is the sire (co)variance matrix. Similar to the PK analysis, the sire variance estimate was transformed algebraically into $\sigma_{a}^{2}$ by multiplying the sire variance by four. The heritability $\left(h_{t}^{2}\right)$ for a given time point was then estimated as:

$$\begin{array}{l} h_{t}^{2}=\;\frac{\sigma_{a}^{2}\;}{\sigma_{sire\text{\_}t}^{2}+\sigma_{batch}^{2}+\sigma_{e}^{2}\;}. \end{array}$$

Lastly, the change in heritability for the fenbendazole metabolite model across time points is a function of not only the change in sire variance across time points, but also the change in residual variance.

## Results

### Pharmacokinetic parameter model

The heritability (± SE) estimates for PK parameters for the parent drug and metabolite for fenbendazole and flunixin meglumine are outlined in Table 3. For the parent drug, the mean (min-max) heritability across PK parameters was 0.15 (0.01–0.28) and 0.31 (0.04–0.58) for fenbendazole and flunixin meglumine, respectively. For the metabolite, the mean heritability across PK parameters was 0.18 (0.06–0.36) and 0.40 (0.18–0.61) for fenbendazole and flunixin meglumine, respectively. The heritability estimation error (i.e., h^2^ ± *SE*) included zero across the majority of PK parameters. The heritability estimate for T_max_ for the metabolite for flunixin meglumine was outside the bounds and therefore was not shown. Least-squares means for fenbendazole and flunixin meglumine and its associated metabolite by breed and sex are outlined in Tables S1, S2, respectively. The impact of sex on a PK parameters never reached the significance level (*P* > 0.05) for fenbendazole, although was trending toward significance (*P* = 0.063) for AUC_0 → ∞_ for the parent drug of flunixin meglumine. Similarly, the impact of breed on PK parameters never reached the significance level for fenbendazole, but was trending toward significance for MRT for the parent drug flunixin meglumine.

**Table 3:** Heritability (± SE) across pharmacokinetic (PK) parameters by drug.

**Compound**	**PK Parameter^a^**	**Heritability (**± **SE)**	
		**Fenbendazole**	**Flunixin meglumine**
Drug	T_1/2_	0.16 ± 0.36	0.20 ± 0.37
	Cl	0.01 ± 0.26	0.42 ± 0.39
	AUC_0 → ∞_	0.13 ± 0.38	0.32 ± 0.35
	MRT	0.16 ± 0.38	0.04 ± 0.28
	Vd_ss_	0.28 ± 0.44	0.58 ± 0.44
Metabolite	AUC_0 → ∞_	0.36 ± 0.41	0.61 ± 0.42
	C_max_	0.13 ± 0.25	0.18 ± 0.25
	T_max_	0.06 ± 0.36	–

a The PK parameters were half-life (T1/2; h), clearance (Cl; L/h/kg), area under the plasma concentration-time curve from time zero to to infinity (AUC_0 → ∞_; h^*^μg/mL), mean residence time (MRT; h), volume of distribution at steady state (Vdss; L/kg), peak concentration (Cmax; μg/mL), and time at which maximum concentration occurs (Tmax; h).

### Random regression model

The heritability (± SE) across time points for the parent drug and metabolite for fenbendazole and flunixin meglumine is outlined in Table 4. For the parent drug, the mean heritability across time was 0.27 and 0.14 for fenbendazole and flunixin meglumine, respectively. Furthermore, across both drugs the heritability increased slightly at 4 and 2–3 h post drug administration for fenbendazole and flunixin meglumine, respectively, which corresponded to an increase in the phenotypic concentration of the drug in the plasma. It should be noted that the change in heritability was not a function of the change in additive genetic variance, but was instead due to a change in the proportion of the variance explained by the model. For the metabolite, the mean heritability across time was higher than its associated parent drug and was 0.60 and 0.44 for fenbendazole and flunixin meglumine, respectively. The population mean concentration curve and sire deviations from the curve along with variance components estimates by hour are shown in Figures S2–S5 for the fenbendazole parent drug, fenbendazole metabolite, flunixin meglumine parent drug and flunixin meglumine metabolite, respectively. The shape of the concentration curve for fenbendazole did not depend on the sex of the animal for the parent drug, but had a tendency to depend on the sex (*P* = 0.078) for the metabolite. Conversely, for flunixin meglumine, sex had an impact on the shape of the concentration curve for the parent drug (*P* = 0.017), but did not have an impact on the metabolite concentration profile. The predicted shape of the concentration curve by sex for the fenbendazole metabolite and flunixin meglumine parent drug is shown in Figure S1. Across both drugs and within the parent drug and metabolite, the concentration of the compound across time was not impacted by breed.

**Table 4:** Heritability (± SE) across time since the drug was administered for fenbendazole and flunixin meglumine parent drug and metabolite concentrations.

**Time (h)^a^**	**Fenbendazole**		**Time**	**Flunixin Meglumine**	
	**Parent Drug**	**Metabolite^b^**		**Parent Drug**	**Metabolite**
0.5	0.451 ± 0.20	0.732	0.5	0.193 ± 0.10	0.312 ± 0.14
1	0.195 ± 0.09	0.611	1	0.118 ± 0.06	0.470 ± 0.21
2	0.097± 0.05	0.675	2	0.167 ± 0.09	0.443 ± 0.20
3	0.110± 0.05	0.549	3	0.162 ± 0.09	0.249 ± 0.12
4	0.311± 0.14	0.479	4	0.122 ± 0.06	0.466 ± 0.21
6	0.181± 0.08	0.599	8	0.125 ± 0.07	0.525 ± 0.23
12	0.333± 0.15	0.355	12	0.081 ± 0.04	0.468 ± 0.21
–	–	–	16	0.142 ± 0.08	0.487 ± 0.22
24	0.225± 0.10	0.783	24	0.106± 0.06	0.550 ± 0.24
48	0.527± 0.23	–	48	0.200 ± 0.11	0.420± 0.19

a *Refers to the hour since drug was administered*.

b *Standard errors were not available*.

## Discussion

The current study has utilized PK parameters or plasma concentrations of the drug or metabolite across time to determine the amount of phenotypic variation explained by genetics. To our knowledge heritability estimates on drug metabolism parameters have not been estimated for any drug in livestock populations, although multiple twin—(Matthaei et al., 2015, 2016) or repeated drug administration—(Kalow et al., 1999; Ozdemir et al., 2000) based genetic parameter estimation studies have been conducted in humans. A major advantage of estimating genetic components for pharmacokinetic parameters in livestock is the ability to exploit the large full-sib and half-sib structure of these populations. Furthermore, males are mated to a large number of females and those females live across a large variety of environmental conditions. As a result, more precise estimates of heritability can be obtained that are not biased by environmental (common and temporary) or dominance and epistatic effects, which are difficult to completely account for in twin studies (Falconer and Mackay, 1996), making livestock, and particularly swine, a potentially viable model for human pharmacogenetics/genomics studies.

In humans, the reported proportion of phenotypic variation for a PK parameter explained by genetics across multiple drugs ranges from minimal (Kalow et al., 1999; Matthaei et al., 2016) to as much as 0.91 (95 percent CI 0.87–0.96) of the phenotypic variation (Matthaei et al., 2015). The average PK heritability estimates across both drugs in the current study was 0.23 and 0.27 for the parent drug and metabolite, respectively, albeit across most PK heritability point estimates the standard error did contain 0 or 1.

Given the large sample size of the current study, models that characterize the drug concentration curve across time were also investigated. The average heritability estimate for the parent drug across time for the drug concentration curve was similar (i.e., 0.20) to the point estimate derived from the PK model. The average heritability estimate for the metabolite across time was 0.51, which was higher than the point estimate derived from the PK model. More importantly, the heritability estimation error (i.e., h^2^ ± *SE*) of the drug concentration curve were much lower and did not contain 0 or 1, which suggest a sizable additive genetic component for the drug concentration curves. Lastly, the sire model used in the current study allows for the prediction of the drug concentration curve of a given sire based on drug concentrations in the associated progeny. As shown in Figures S2–S6, there are differences across sires in the predicted drug concentration curves.

The initial study by Howard et al. (2014), utilized a subset of the population (i.e., Batch 1–4) in order to determine if breed and/or sex differences exist across PK parameters for fenbendazole and flunixin meglumine. The previous study found differences across breeds for flunixin meglumine (*P* < 0.05; Cl, Vd_ss_) and oxfendazole (*P* < 0.05, AUC_0 → ∞_). Given the larger sample size and a different objective in the current study, an additional sire effect was included in the model compared to that used in Howard et al. (2014). As a result, a proportion of the previously estimated breed effect is now captured by the sire effect. This was confirmed by removing the random effect of sire out of the PK based heritability model which, as expected, resulted in greater proportion of the PK parameters being affected by breed.

As discussed in Howard et al. (2015), the use of pigs as an animal model in human medicine allows for one to efficiently generate lines within different drug metabolism profiles. This was further verified in the current study given the moderate heritability for the metabolism of fenbendazole and flunixin meglumine observed in the current resource population. Furthermore, the environmental sources of variation can be effectively controlled along with a planned mating design to understand the variability across animals in how they metabolize a given drug more precisely, which is not feasible with human clinical trials. Pharmacogenomics research not only allows for greater insight into human drug metabolism, but also in livestock. A greater understanding of the impact of individuals/populations with different drug concentration (i.e., high vs. low drug clearance) profiles on disease progression and prevalence allows for drug selection to be more precise, as well as the possibility to select individuals that have a more uniform drug metabolism profile. This could in turn be employed to standardize treatment, increase drug efficacy and reduce the chances of possible residues in edible tissues.

The current study estimated the proportion of phenotypic variation in metabolizing fenbendazole and flunixin meglumine that is due to additive genetics. Models to estimate heritability based on PK parameters or the observed plasma drug concentration across time were utilized. Across both types of models a moderate heritability was estimated. Furthermore, the model that utilized the plasma drug concentration across time vs. the PK parameters resulted in more precise estimates.

## Author contributions

JH performed the statistical analysis. RB, JB, and JY performed the pharmacokinetic analysis. CM, RB, and MA designed and conceived the experiment. JH wrote the first draft of the paper. All authors read and approved the final manuscript.

### Conflict of interest statement

The authors declare that the research was conducted in the absence of any commercial or financial relationships that could be construed as a potential conflict of interest.

## References

[B1] Buur J. L. Baynes R. E. Smith G. Riviere J. E. (2006). Pharmacokinetics of flunixin meglumine in swine after intravenous dosing. J. Vet. Pharmacol. Ther. 29, 437–440. 10.1111/j.1365-2885.2006.00788.x 16958790

[B2] Falconer D. S. Mackay T. F. C. (1996). Introduction to Quantitative Genetics, 4th Edn. New York, NY: Longman Scientific and Technical.

[B3] Gilmour A. R. Gogel B. J. Cullis B. R. Thompson R. (2009). ASReml User Guide Release 3.0. Hemel Hempstead: VSN International Ltd.

[B4] Howard J. T. Baynes R. E. Brooks J. D. Yeatts J. L. Bellis B. Ashwell M. S.. (2014). The effect of breed and sex on sulfamethazine, enrofloxacin, fenbendazole and flunixin meglumine pharmacokinetic parameters in swine. J. Vet. Pharmacol. 37, 531–541 10.1111/jvp.12128 24731191

[B5] Howard J. T. Ashwell R. E. Baynes J. D. Brooks J. L. Yeatts C. Maltecca C. (2017). Gene co-expression network analysis identifies porcine genes associated with variation in metabolizing fenbendazole and funixin meglumine in the liver. Sci. Rep. 7:1357. 10.1038/s41598-017-01526-5 28465592 PMC5430975

[B6] Howard J. T. O'Nan A. T. Maltecca C. Baynes R. E. Ashwell M. S. (2015). Differential gene expression across breed and sex in commercial pigs administered fenbendazole and flunixin meglumine. PLoS ONE 10:e0137830 10.1371/journal.pone.0137830 26366864 PMC4569569

[B7] Kalow W. Endrenyi L. Tang B. (1999). Repeat administration of drugs as a means to assess the genetic component in pharmacological variability. Pharmacology 58, 281–284. 10325572 10.1159/000028292

[B8] Landers T. F. Cohen B. Wittum T. E. Larson E. L. (2012). A review of antibiotic use in food animals: perspective, policy, and potential. Public Health Rep. 127, 4–22. 10.1177/003335491212700103 22298919 PMC3234384

[B9] Lin Z. Vahl C. I. Riviere J. E. (2016). Human food safety implications of variation in food animal drug metabolism. Sci. Rep 6:27907. 10.1038/srep27907 27302389 PMC4908408

[B10] Matthaei J. Brockmöller J. Tzvetkov M. V. Sehrt D. Sachse-Seeboth C. Hjelmborg J. B.. (2015). Heritability of metoprolol and torsemide pharmacokinetics. Clin. Pharmacol. Ther. 98, 611–621. 10.1002/cpt.258 26344676

[B11] Matthaei J. Tzvetkov M. V. Gal V. Sachse-Seeboth C. Sehrt D. Hjelmborg J. B.. (2016). Low heritability in pharmacokinetics of talinolol: a pharmacogenetic twin study on the heritability of the pharmacokinetics of talinolol, a putative probe drug of MDR1 and other membrane transporters. Genome Med. 8, 119. 10.1186/s13073-016-0372-2 27825374 PMC5101708

[B12] Ozdemir V. Kalow K. Tang B. K. Paterson A. D. Walker S. E. Endrenyi L.. (2000). Evaluation of the genetic component of variability in CYP3A4 activity: a repeated drug administration method. Pharmacogenetics 10, 373–388. 10.1097/00008571-200007000-00001 10898107

[B13] Pairis-Garcia M. D. Karriker L. A. Johnson A. K. Kukanich B. Wulf L. Sander S.. (2013). Pharmacokinetics of flunixin meglumine in mature swine after intravenous, intramuscular and oral administration. BMC Vet. Res. 9:165. 10.1186/1746-6148-9-165 23941181 PMC3751365

[B14] Petersen M. B. Friis C. (2000). Pharmacokinetics of fenbendazole following intravenous and oral administration to pigs. Am. J. Vet. Res. 61, 573–576. 10.2460/ajvr.2000.61.573 10803655

