# Supplementary material for: Genetic Parameter Estimates for Metabolizing Two Common Pharmaceuticals in Swine
Source: Front Genet. 2018 Feb 13;9:40. doi: 10.3389/fgene.2018.00040 (PMC5816749; doi:10.3389/fgene.2018.00040)
Supplement: Supplementary file 1 [file DataSheet1.DOCX]

**Table S1.** Fenbendazole and oxfendazole pharmacokinetic parameter least-squares means by breed and sex.

|  |  | Breed^1^ LS Means±SE | | | | | Sex LS Means±SE | | |
| --- | --- | --- | --- | --- | --- | --- | --- | --- | --- |
|  | PK^2^ | D | H | L | Y | P-value | Female | Male | P-value |
| Drug | T_1/2_ | 17.45±2.45 | 16.47±2.67 | 13.12±2.55 | 12.61±2.62 | 0.56 | 14.22±1.80 | 15.60±1.59 | 0.49 |
|  | Cl | 0.25±0.04 | 0.27±0.04 | 0.33±0.04 | 0.31±0.04 | 0.51 | 0.29±0.03 | 0.28±0.03 | 0.90 |
|  | AUC_0→∞_ | 6.32±0.88 | 5.77±0.96 | 5.36±0.92 | 4.20±0.95 | 0.47 | 5.32±0.65 | 5.50±0.58 | 0.83 |
|  | MRT | 16.68±2.91 | 15.89±3.17 | 13.49±3.03 | 13.11±3.11 | 0.81 | 14.17±2.14 | 15.42±1.88 | 0.65 |
|  | Vd_ss_ | 2.87±0.38 | 3.20±0.41 | 2.67±0.39 | 2.79±0.40 | 0.83 | 2.81±0.26 | 2.95±0.23 | 0.68 |
| Metabolite | AUC_0→∞_ | 6.59±0.44 | 7.06±0.46 | 7.41±0.47 | 7.46±0.46 | 0.56 | 7.33±0.29 | 6.93±0.25 | 0.29 |
|  | C_max_ | 0.45±0.02 | 0.48±0.02 | 0.48±0.02 | 0.45±0.02 | 0.46 | 0.46±0.01 | 0.47±0.01 | 0.42 |
|  | T_max_ | 3.28±0.15 | 3.32±0.15 | 3.32±0.16 | 3.19±0.16 | 0.93 | 3.19±0.11 | 3.37±0.09 | 0.21 |

^1^ Refers to breed and D = Duroc; H = Hampshire; L=Landrace; Y=Yorkshire.

^2^ The pharmacokinetic (PK) parameters were half-life (T_1/2_; h), clearance (Cl; L/h/kg), area under the plasma concentration-time curve from time zero to infinity (AUC_0→∞_; h*$\mu$g/ml), mean residence time (MRT; h), volume of distribution at steady state (Vd_ss;_ L/kg), peak concentration (C_max_; $\mu$g/ml) and time at which maximum concentration occurs (T_max_; h).

**Table S2**. Flunixin meglumine and 5-hydroxy flunixin pharmacokinetic parameter least-squares means by breed and sex.

|  |  | Breed^1^ LS Means±SE | | | | | Sex LS Means±SE | | | |
| --- | --- | --- | --- | --- | --- | --- | --- | --- | --- | --- |
|  | PK^2^ | D  (n=8;8 )^3^ | H  (n=9;9 )^3^ | L  (n=14;13)^3^ | Y  (n=10;10)^3^ | P-  value | Female  (n=21;21 )^3^ | Male  (n=20;19 )^3^ | P-  value |  |
| Drug | T_1/2_ | 6.57±0.48 | 6.24±0.47 | 6.80±0.45 | 6.73±0.49 | 0.84 | 6.45±0.31 | 6.71±0.29 | 0.53 |  |
|  | Cl | 0.12±0.01 | 0.13±0.01 | 0.11±0.01 | 0.13±0.01 | 0.37 | 0.12±0.005 | 0.12±0.004 | 0.17 |  |
|  | AUC_0→∞_ | 27.42±1.78 | 27.20±1.81 | 28.22±1.66 | 27.59±1.82 | 0.98 | 26.28±1.01 | 28.94±1.03 | 0.06 |  |
|  | MRT | 3.98±0.25^a^ | 3.88±0.24^a^ | 4.07±0.23^a^ | 3.12±0.26^b^ | 0.09 | 3.59±0.17 | 3.94±0.16 | 0.14 |  |
|  | Vd_ss_ | 0.44±0.04 | 0.50±0.04 | 0.42±0.04 | 0.37±0.04 | 0.30 | 0.43±0.02 | 0.42±0.02 | 0.71 |  |
| Metabolite | AUC_0→∞_ | 0.73±0.10 | 0.83±0.11 | 0.90±0.10 | 0.85±0.10 | 0.74 | 0.81±0.06 | 0.85±0.05 | 0.57 |  |
|  | C_max_ | 0.21±0.02 | 0.20±0.02 | 0.22±0.02 | 0.26±0.02 | 0.15 | 0.22±0.01 | 0.22±0.01 | 0.71 |  |

^a,b^ Least-square means within a row and effect with different superscripts differ (P<0.05).

^c,d^ Least-square means within a row and effect with different superscripts differ (P<0.10).

^1^ Refers to breed and D = Duroc; H = Hampshire; L=Landrace; Y=Yorkshire.

^2^ The pharmacokinetic (PK) parameters were half-life (T_1/2_; h), clearance (Cl; L/h/kg), area under the plasma concentration-time curve from time zero to infinity (AUC_0→∞_; h*$\mu$g/ml), mean residence time (MRT; h), volume of distribution at steady state (Vd_ss;_ L/kg), peak concentration (C_max_; $\mu$g/ml).

^3^ Refers to the number of animals within each breed and sex, with the first and second number pertaining to the drug and metabolite, respectively.

**Figure S1.** Predicted shape of the concentration curve by sex for the fenbendazole metabolite and flunixin meglumine parent drug.


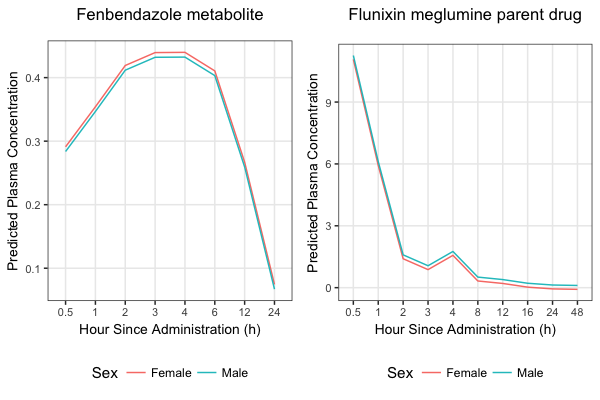


**Figure S2**. Predicted population (black line) and sire (colored lines) concentration^1^ across time and proportion of phenotypic variance explained for the random terms in the model for fenbendazole parent drug.


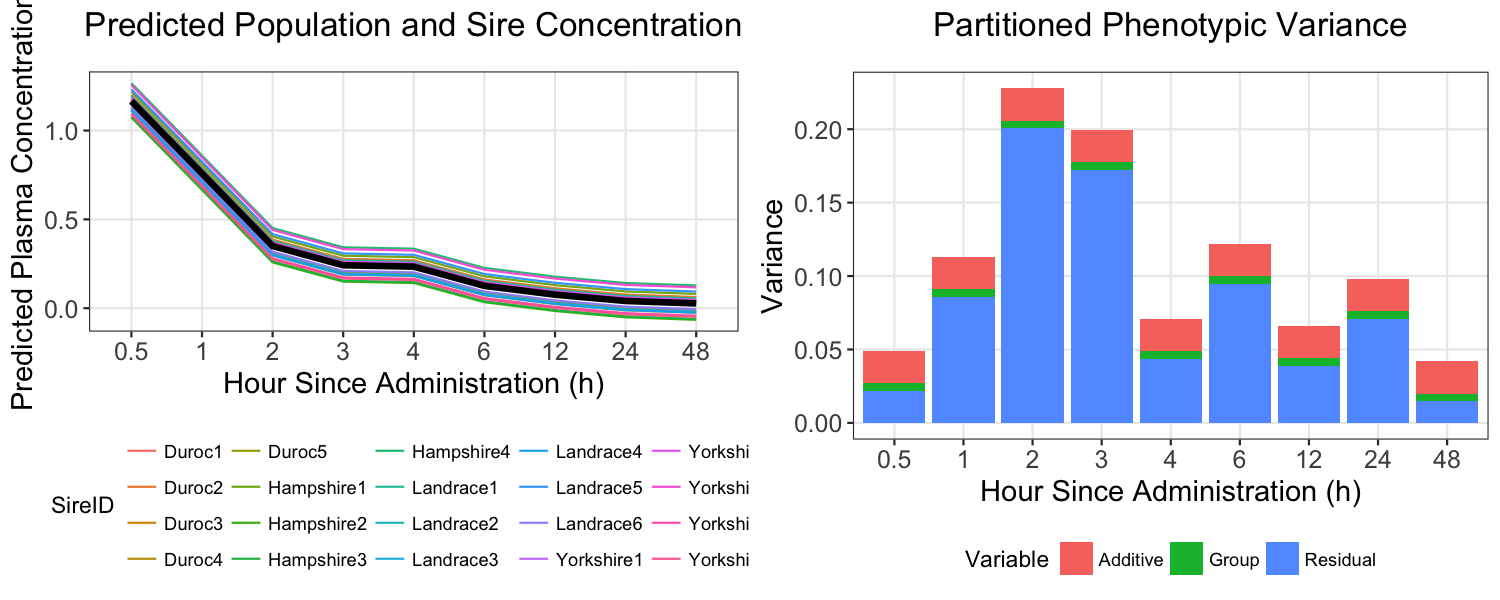


^1^ Predicted concentrations were in some cases less than zero for time points past 12 hours since drug administration as a result of a low concentration of the drug and the prediction error of the estimate.

**Figure S3**. Predicted population (black line) and sire (colored lines) concentration across time and proportion of phenotypic variance explained for the random terms in the model for fenbendazole metabolite.


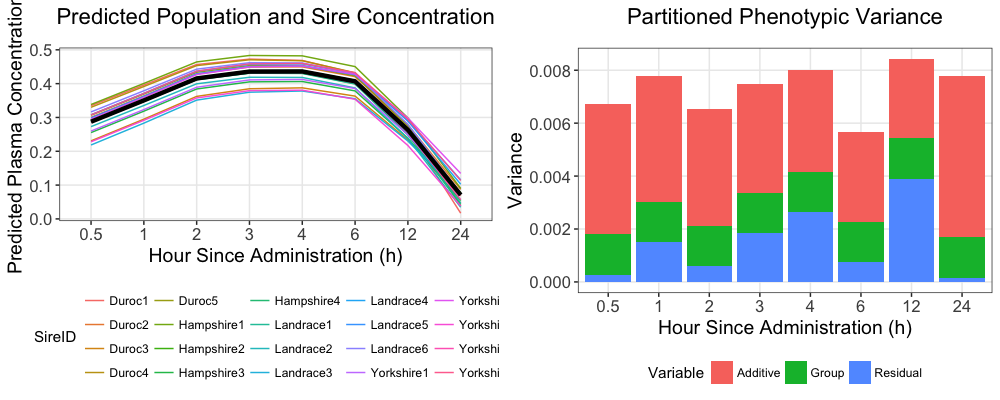


**Figure S4**. Predicted population (black line) and sire (colored lines) concentration^1^ across time and proportion of phenotypic variance explained for the random terms in the model for flunixin meglumine parent drug.


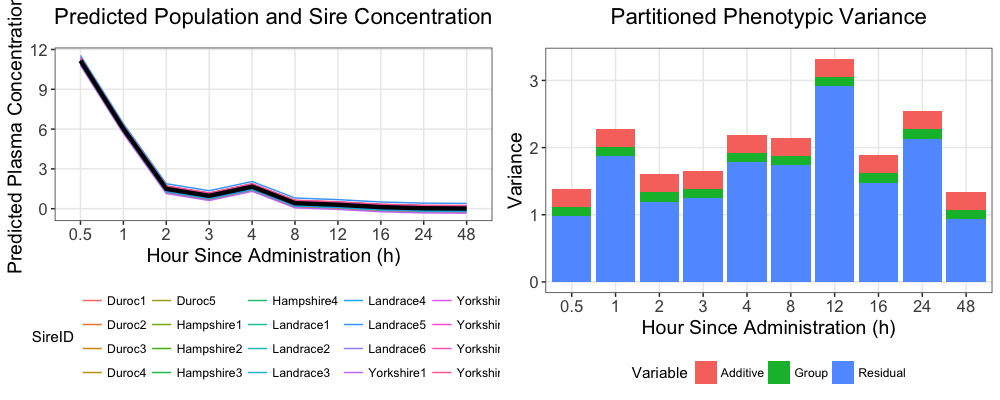


^1^ Predicted concentrations were in some cases less than zero for time points past 12 hours since drug administration as a result of a low concentration of the drug and the prediction error of the estimate.

**Figure S5**. Predicted population (black line) and sire (colored lines) concentration^1^ across time and proportion of phenotypic variance explained for the random terms in the model for flunixin meglumine metabolite.


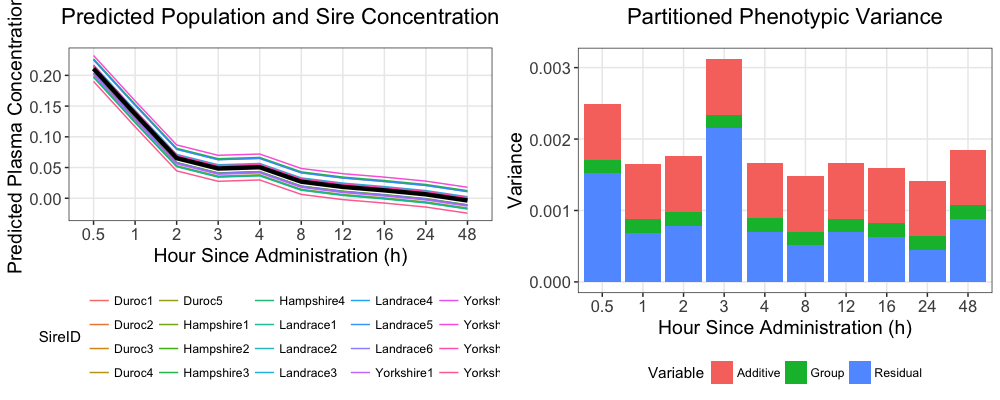


^1^ Predicted concentrations were in some cases less than zero for time points past 12 hours since drug administration as a result of a low concentration of the drug and the prediction error of the estimate.
